# A live attenuated *Salmonella* Typhimurium vaccine dose and diluent have minimal effects on the caecal microbiota of layer chickens

**DOI:** 10.3389/fvets.2024.1364731

**Published:** 2024-04-15

**Authors:** Samiullah Khan, Andrea R. McWhorter, Daniel M. Andrews, Gregory J. Underwood, Robert J. Moore, Thi Thu Hao Van, Richard K. Gast, Kapil K. Chousalkar

**Affiliations:** ^1^School of Animal and Veterinary Sciences, The University of Adelaide, Roseworthy, SA, Australia; ^2^Bioproperties Pty Ltd, Ringwood, VIC, Australia; ^3^School of Science, RMIT University, Bundoora, VIC, Australia; ^4^U. S. National Poultry Research Center, USDA Agricultural Research Service, Athens, GA, United States

**Keywords:** *Salmonella* Typhimurium vaccine, layer chicks, poultry production, salmonellosis, gut microbiota

## Abstract

Among the *Salmonella* reduction strategies in poultry production, one option is to use a *Salmonella* vaccine. The aim of vaccinating layer flocks is to reduce the shedding of wild-type *Salmonella* in the poultry environment, thereby reducing the contamination of poultry products (eggs and meat). Nutritive diluent and a higher dose of vaccine may enhance its colonization potential in the gut of chickens. In this study, a commercially available live attenuated vaccine (Vaxsafe^®^ ST) was reconstituted in different media and delivered orally to day-old chicks at three different doses (10^7^, 10^8^, and 10^9^ CFU/chick). Gut colonization of the vaccine strain and the effects of vaccination on gut microbiota were assessed in commercial-layer chickens. The vaccine diluent and dosage minimally affected microbiota alpha diversity. Microbiota beta diversity was significantly different (P < 0.05) based on the vaccine diluent and dose, which indicated that the vaccinated and unvaccinated chickens had different gut microbial communities. Differences were noted in the abundance of several genera, including *Blautia, Colidextribacter, Dickeya, Enterococcus, Lactobacillus, Pediococcus*, and *Sellimonas*. The abundance of *Colidextribacter* was significantly lower in chickens that received vaccine reconstituted in Marek's and water diluents, while *Lactobacillus* abundance was significantly lower in the water group. The highest vaccine dose (10^9^ CFU/chick) did not significantly alter (*P* > 0.05) the abundance of microbial genera. Chicken age affected the microbiota composition more significantly than the vaccine dose and diluent. The abundance of *Lactobacillus, Blautia, Caproiciproducens, Pediococcus*, and *Colidextribacter* was significantly higher on day 14 compared with day 7 post-vaccination. The *Salmonella* Typhimurium vaccine load in the caeca was not significantly affected by diluent and vaccine dose; however, it was significantly lower (*P* < 0.0001) on day 14 compared with day 7 post-vaccination. Overall, the *S*. Typhimurium vaccine minimally affected the gut microbiota structure of layer chicks, whereas changes in microbiota were more significant with chicken age.

## Introduction

Pathogenic serotypes of *Salmonella* associated with food-producing animals cause salmonellosis in humans if contaminated products are consumed. According to the World Health Organization (WHO), *Salmonella* is one of four key causes of diarrheal diseases globally. In European Union member countries, a total of 50,817 human salmonellosis cases were reported in 2021 (1). In Australia, in 2021, the National Notifiable Disease Surveillance System documented a total of 10,828 reported cases of human salmonellosis. Chicken meat and eggs are a significant source of human salmonellosis (2). Therefore, strategies to reduce *Salmonella* contamination at the farm level are one way to reduce human salmonellosis.

Among the *Salmonella* reduction strategies at the farm level, vaccination of flocks plays a pivotal role. Vaccinated flocks shed lower levels of *Salmonella*, and this results in reduced contamination of eggs and meat during egg grading or meat processing (3). In Australia, many commercial layer breeders, broiler breeders, and commercial layers are vaccinated with a live, attenuated *Salmonella* Typhimurium vaccine, Vaxsafe^®^ ST (Bioproperties Pty Ltd). Vaxsafe ST is an *aroA* mutant, freeze-dried vaccine that is normally reconstituted in sterile water for spray and oral administration. The *aroA* mutation disrupts the shikimate biochemical pathway, thus disrupting the production of essential aromatic amino acids, which prevents growth in the host as these compounds are not freely available. Previous studies of Vaxsafe ST in chickens showed that it was partially effective in protecting chickens from wild-type *Salmonella* infection (4, 5). The other forms of *Salmonella* vaccines, autogenous or killed vaccines, are not commonly used in the Australian poultry industry. A study comparing the effects of live, attenuated versus multivalently killed vaccines showed that the killed vaccine provided short-term protection, whereas the injected live, attenuated vaccine produced longer-lasting protection (4). The limitation of autogenous vaccines is that they must be administered multiple times for priming and the generation of long-term immune responses. Multiple administrations of an injected vaccine make it impractical, particularly considering chicken flock size and the associated labor cost. The live, attenuated vaccine registered in Australia, Vaxsafe ST, is administered on day 1 via coarse spray, at weeks 2 to 4 via drinking water, and at week 12 of chicken age through intramuscular injection, a vaccination protocol that is economically feasible and widely adopted. For intramuscular administration at 12 weeks of chicken age, this vaccine is either reconstituted in water or Marek's diluent.

In recent years, many studies have been undertaken to understand and define the beneficial role of gut microbiota in the competitive exclusion of pathogens and how this is influenced by variations in composition, host environment, and treatments. In chickens, differences in the gut microbiota of broilers and layers (6) indicate the role of genotype and diet. In layer chickens, gut microbiota varies with age and gut segment, where there are more taxonomical complex microbiota present in the caecum compared with the ileum (7). Days 1 to 3 of chicken age are critical for microbiota development, whereas at around day 7, most of the taxa found in mature birds are already present; however, fluctuations in the abundance of microbial community members continue for several weeks (8).

Gut microbiota can be influenced positively or negatively, depending upon the triggering factor and its associated level. In chickens, among different segments of the gut, caeca contain the highest diversity and most dense populations of microbiota (9). Generally, stress conditions, infectious agents, and the use of antibiotics can alter the gut microbiota. For example, the use of dietary antibiotics reduced alpha diversity (richness), beta diversity, and abundance of the caecal microbiota in broilers (10). In laying hens, heat stress has been shown to reduce Firmicutes and increase Bacteroidetes in the feces, leading to the perturbation of the gut microbiota and its functions (11). Infectious agents can have profound negative effects on chicken gut microbiota, as seen in Marek's disease (12), infectious bronchitis (13), campylobacteriosis (14), salmonellosis (15), necrotic enteritis (16), and avian influenza (17).

Unlike infectious pathogens, the vaccines used to protect against them have not been extensively tested for microbiota interactions for many poultry vaccines. A study in broiler chickens suggested that coccidiosis vaccination positively influenced the population of *Lactobacillaceae, Enterobacteriaceae, Clostridiaceae*, and *Streptococcaceae* in caeca at 21 days of age (18). An oral administration of a recombinant *S*. Typhimurium vaccine in 4-day-old White Leghorn chickens significantly altered the alpha diversity (richness) and beta diversity of the caecal microbiota analyzed at week 5 of chicken age (19).

Since Vaxsafe ST is prepared by partial disruption of the *aroA* gene, the vaccine is unable to synthesize some aromatic compounds. Bacteria grown in nutritive broth may increase the expression of genes from *Salmonella* pathogenicity island-1 that facilitate attachment and invasion into intestinal epithelial cells. Multiple *Salmonella* serovars grown statically on agar and suspended in saline exhibit markedly lower invasion capacities than the same strains grown in nutrient-rich broth (20, 21). Therefore, we hypothesized that reconstituting Vaxsafe ST in nutritive diluent would enhance its colonization ability in the gut of chickens. Marek's diluent was selected as it is used in the poultry industry for Vaxsafe ST reconstitution for intramuscular injection at week 12 and is also used for reconstituting Marek's disease vaccine. Buffered peptone water (BPW) diluent was also used, as its composition is very similar to Marek's diluent. To compare the nutritive diluents, sterile water was used as a control. Additionally, multiple doses of Vaxsafe ST were used to evaluate whether a higher dose would lead to a significant increase in vaccine colonization in the gut. The main objectives of the current study were to understand the effects of vaccine diluent and vaccine (Vaxsafe ST) dosage on vaccine colonization in the caeca and the host caecal microbiota in commercial layer chickens vaccinated at day old, a time when the gut microbiota is rapidly changing.

## Materials and methods

### Ethics statement

The Animal Ethics Committee at the University of Adelaide approved all work (approval number S-2017-080) in accordance with the guidelines specified in “Australian code for the care and use of animals for scientific purposes, 8th edition (2013)”. The study followed the ARRIVE guidelines required for *in vivo* experiments (22).

### Hatching and rearing of layer chickens

Mixed-sex Isa-Brown layer chickens were hatched and reared (*n* = 192) at the School of Animal and Veterinary Sciences, Roseworthy Campus, The University of Adelaide, as per standard procedures detailed in the Isa-Brown Management Guide. Different treatment groups were reared in floor pens (55 cm wide × 125 cm long) in separate rooms in a small animal research facility. Birds were divided into 12 treatment groups, and at each sampling timepoint, a minimum of 6 chickens were humanely euthanized by cervical dislocation. The mesh in each pen was covered with chick paper (205 GSM). *Salmonella*-free status of the hatched chickens was confirmed through the culturing of meconium samples (*n* = 4) from the incubator (Maru 190 Deluxe Cabinet), as previously described (15). The details of the treatment groups are provided in Table 1.

**Table 1:** Treatment groups, vaccine dose and diluents used in this study.

**Diluent**	**Treatment group**	**Vaccine dose (CFU)**
Marek's	Marek's 10^7^ CFU	10^7^
	Marek's 10^8^ CFU	10^8^
	Marek's 10^9^ CFU	10^9^
	Marek's control	Nil
BPW	BPW 10^7^ CFU	10^7^
	BPW 10^8^ CFU	10^8^
	BPW 10^9^ CFU	10^9^
	BPW control	Nil
Water	Water 10^7^ CFU	10^7^
	Water 10^8^ CFU	10^8^
	Water 10^9^ CFU	10^9^
	Water control	Nil

### Vaccine reconstitution and chicken vaccination

Vials of Vaxsafe ST (Bioproperties) were either reconstituted in 1 mL of Marek's diluent, buffered peptone water, or water. For Marek's diluent, the reconstituted vaccine vial (containing 10^10^ CFU/mL) was 10-fold serially diluted in the same diluent to achieve 10^9^ and 10^8^ CFU per ml. The process was repeated to prepare vaccine dilutions in BPW and water diluents. The vaccine manufacturer's recommended dose is 10^7^ CFU per chicken. The higher vaccine doses (10^9^ and 10^8^ CFU/chick) were included in the study to understand if they enhanced vaccine colonization ability in the gut. On day 1 post-hatch, individual chicken in the respective treatment groups received 100 μl doses of the respective reconstituted vaccine orally. Each bird was orally vaccinated to ensure uniform administration of the vaccine. Unvaccinated chickens received 100 μl of the sterile diluents. Chickens were euthanized on days 7 and 14 for caecal microbiota analysis and vaccine load quantification in the caecal contents. Caecal contents were collected and frozen at−80°C until used for DNA extraction.

### Total DNA extraction from caecal contents

Total DNA was extracted from 192 luminal caecal content samples using a modified protocol for the QIAamp FAST DNA Stool Mini Kit (Qiagen). Caecal contents were collected from birds at each sampling point. Briefly, approximately 200 mg of caecal contents per sample was weighed into a 1.5-ml safe-lock tube and into each sample 700 μl of InhibitEx Buffer was added. For maximum lysis of microbial cells, a mixture of glass beads (acid-washed ≤ 106 μm and 425–600 μm; Sigma Aldrich) was added to the samples and homogenized in a bullet blender (Next Advances) for 5 min at speed 10. The samples were processed for DNA extraction as previously described (23). The quantity (average 88 ng/μl) and purity (average 260/280 value of 1.90 and 260/230 value of 1.70) of each DNA sample were tested to ensure that they were suitable for sequencing and qPCR.

### 16s rRNA metagenome sequencing and data analysis

All the samples were PCR-amplified for 16S rRNA gene analysis. The V3-V4 regions were amplified with Q5 high-fidelity polymerase (New England Biolabs) using the 338F (5′-ACTCCTACGGGAGGCAGCAG-3′) and 806R (5′-GGACTACHVGGGTWTCTAAT-3′) primer pairs as previously described (24) and sequenced on an Illumina MiSeq instrument using a 2 × 300 bp paired-end kit. The sequence data were demultiplexed with the onboard Illumina software, and the analysis was performed in Quantitative Insights into Microbial Ecology 2 (QIIME2) (25). Quality filtering, denoising, and chimera removal were performed using Dada2 (26) as a QIIME2 plugin with all recommended parameters, and the sequences were grouped into amplicon sequence variants (ASVs). Taxonomy was assigned using the SILVA v138.1 database (27). The ASV frequency table was loaded into the online MicrobiomeAnalyst analysis system for data normalization using the cumulative sum scaling (CSS) option, and the data were analyzed using alpha diversity, beta diversity, linear discriminant analysis effect size (LEfSe), and non-parametric tests, as per the recommendations of the online software (28).

### Vaccine load determination in cecal contents

#### *Salmonella* Typhimurium vaccine-specific qPCR

Vaxsafe ST-specific qPCR was optimized with the primer pair (F: 5-GGTGTAATTGATCCCCAACG-3 and R: 5-GGTGTAATTGATCCCCAACG-3) designed by the vaccine manufacturing company (Bioproperties, Pty Ltd, Ringwood, Victoria, Australia) that targeted the *aroA* gene and produced a 204-bp product. The PCR was tested for specificity and amplification efficiency using 10-fold serially diluted Vaxsafe ST DNA. The primer pair was also tested against wild-type *S*. Typhimurium PT9 and *Escherichia coli* (chicken isolate) DNA to further test its specificity. The qPCR was performed using the SensiFAST SYBR Hi-ROX Kit (Bioline) in a 20-μl final reaction volume. The reaction volume contained 10 μl SensiFAST buffer, 1 μl each of the forward and reverse primers (10 μM), 2 μl of DNA template, and 6 μl of water. The cycling conditions in a QuantStudio 6 instrument were initial denature at 95°C for 3 min; 40 cycles of annealing at 60°C for 30 s; extension at 72°C for 30 s; a hold stage at 72°C for 5 min; and melting from 60°C to 95°C. The specificity of the primer pair was confirmed by the presence of a single peak in the melt curve analysis and electrophoretic analysis in a 2% agarose gel. The amplification efficiency (%) was calculated using E = -1+10^(−1/slope)^.

### Vaxsafe ST DNA fragment cloning and generation of a standard curve

A freshly generated qPCR product (204-bp amplicon length) of Vaxsafe ST DNA was cloned into a plasmid (pCR4-TOPO) that was inserted into DH5α-T1^R^ chemically competent *E. coli* cells as per the manufacturer's protocol for One Shot Chemical Transformation, TOPO TA Cloning Kit for Sequencing (Invitrogen). The recombinant plasmid was extracted using a PureLink Quick Plasmid Miniprep Kit as per the manufacturer's protocol (Invitrogen). The insertion of the Vaxsafe ST fragment into the plasmid was confirmed by qPCR, melt curve analysis, and running the amplicon on a 2% agarose gel. Vaxsafe ST DNA was used as a positive control. The recombinant plasmid was serially diluted to construct a standard curve for the quantification of vaccine load from caecal contents. The DNA copy number for the recombinant plasmid was calculated from the plasmid DNA concentration and the molecular weight of the plasmid with a Vaxsafe ST fragment insert.

### Vaxsafe ST load in caeca

To quantify the vaccine load from caecal contents, qPCR was performed, in duplicate, on the caecal content DNA of all the samples (*n* = 192). The optimized qPCR was highly sensitive for the quantification of Vaxsafe ST from chicken gut. A 10-fold dilution series of the plasmid was used to construct a standard curve. Vaxsafe ST DNA was included as a positive control, and no-template controls were also used. Samples falling out of the range of the standard curve (Cq > 35) were excluded from the Vaxsafe ST copy number determination.

### Statistical analysis

The *S*. Typhimurium vaccine load (log_10_ DNA copy number) per gram of caecal contents data were analyzed in GraphPad Prism using non-parametric (Mann-Whitney) analysis. The level of significance was determined by PLSD at P < 0.05.

## Results

### Vaccine load in the caeca of chickens

**Figure 1:**
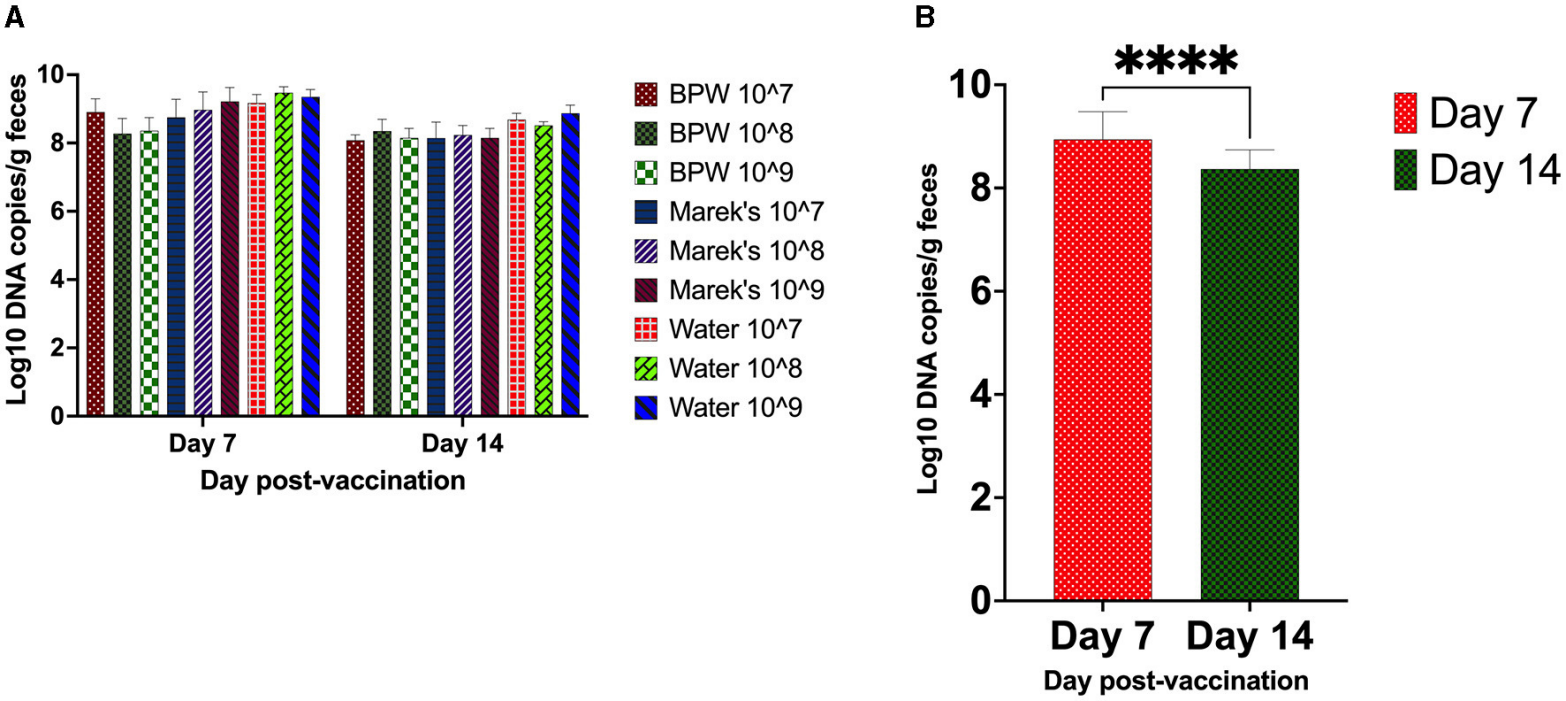
*S*. Typhimurium vaccine (Vaxsafe ST) load in caecal contents of vaccinated chickens. Day-old Isa-Brown layer chickens were vaccinated by reconstituting vaccine in BPW, Marek's diluent, or water and administered at 10^7^, 10^8^, or 10^9^ CFU/chicken. The vaccine load through qPCR was determined on days 7 and 14 post-vaccination. **(A)** Vaccine load is affected by dose and diluent. **(B)** Vaccine load compared on day 7 and day 14 post-vaccination (*P* < 0.0001). DNA extracted from the caecal contents of the unvaccinated groups did not yield any PCR product; therefore, they were excluded from the statistical data analysis. DNA copy number data are log_10_ expressed. In **(B)**, all treatment groups were combined to understand the chicken age's effect on vaccine load. Six chickens from each treatment group were processed on day 7, while up to 11 chickens on day 14 were processed for sample collection. Asterisks (^****^) in **(B)** show a *P* < 0.0001.

The vaccine load from the caecal contents was quantified using qPCR. The optimized qPCR was highly target-specific, with a primer amplification efficiency of 97%. All unvaccinated groups were negative for the vaccine strain. Within each diluent treatment group, there was no significant effect of the vaccine dose on the load of the vaccine in the caecal contents (Figure 1A). The average vaccine load in the caecal contents on day 7 post-vaccination was Log_10_ 8.94, while on day 14, it was Log_10_ 8.37 (Figure 1B).

### The effects of vaccine dosage and diluent on caecal microbiota alpha and beta diversities

**Figure 2:**
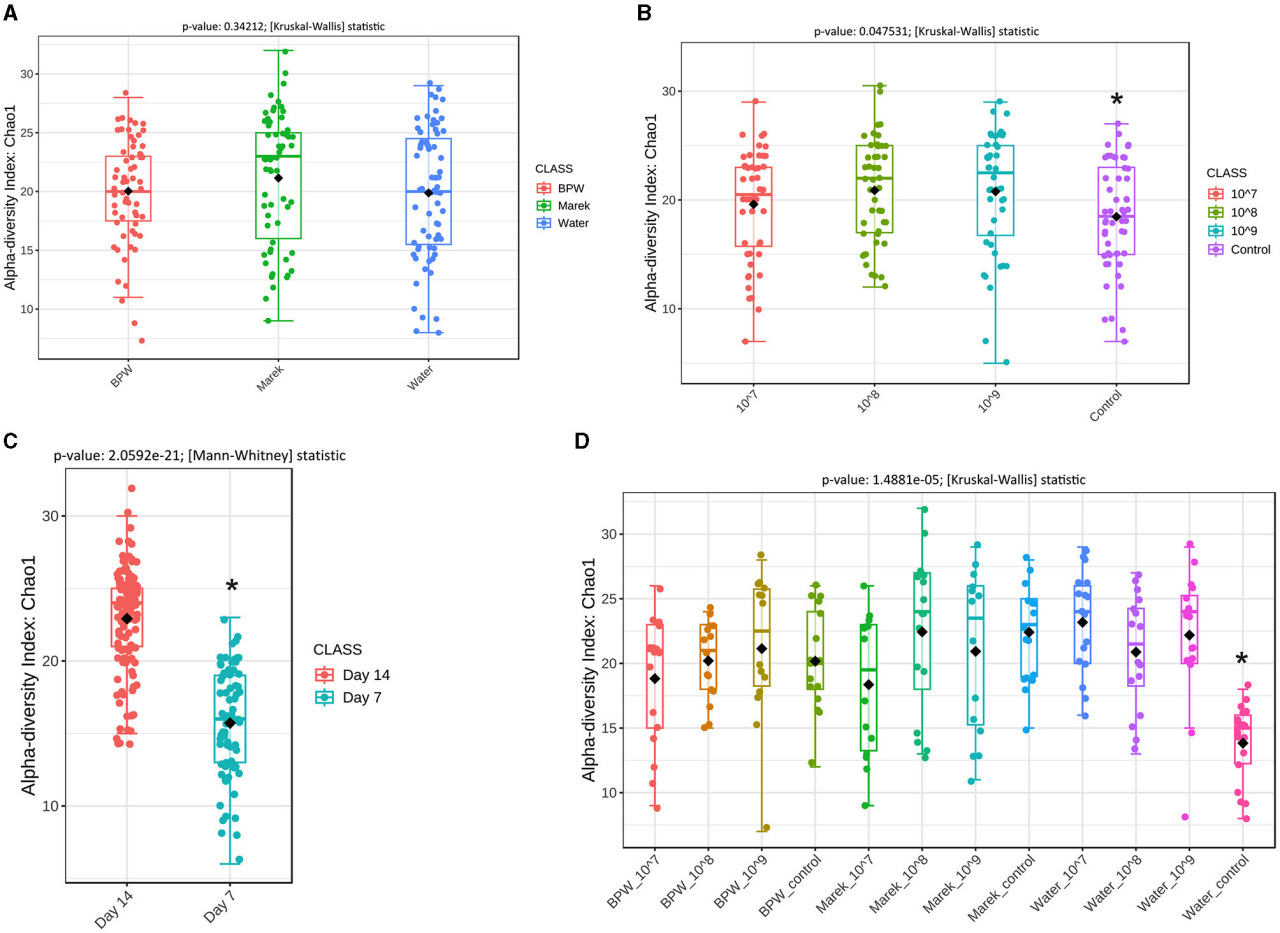
Overall alpha diversity of the caecal microbiota of layer chicks affected by diluent and *Salmonella* Typhimurium vaccine doses. **(A)** Alpha diversity is affected by vaccine diluent (*P* = 0.34212). **(B)** Alpha diversity is affected by vaccine dose (*P* = 0.047531). Data are presented by pooling relevant dose treatment groups together, and the control group represents controls of BPW, Marek's, and water diluents. **(C)** Alpha diversity is affected by age (*P* = 2.0592e-21). All the treatment groups were pooled together based on age. **(D)** Alpha diversity is affected by vaccine dose and diluent in individual treatment groups (*P* = 1.4881e-05). Alpha diversity was by Chao1 using the Mann-Whitney test, while beta diversity was measured by the distance method Bray-Curtis index and ANOSIM statistical method. Diversity was measured at the genus level. Chao1 is a nonparametric method that measures species richness, which refers to the total number of species in a sample. Within each panel graph, an asterisk (*) shows a significant difference. Each treatment group had a minimum of six chickens at each sampling timepoint.

The 16S rRNA gene amplicon sequencing produced 3,646,251 sequences after quality trimming and chimera removal, with an average of 23,224 reads per sample from 157 samples. Alpha diversity measures the community structure of individual samples in terms of how many taxa are present (richness) and the distribution of taxa abundances within a sample (evenness). Prior to administration at three different doses, Vaxsafe ST was reconstituted in buffered peptone water (BPW), Marek's diluent, and water to understand if the diluent and dose had any effects on gut microbiota (Figures 2A–D). There were no significant differences in the alpha diversity of the caecal microbiota of 14-day-old layer chickens vaccinated using different diluents (Figure 2A). However, the chickens vaccinated with 10^8^ and 10^9^ CFU/chicken showed significantly higher alpha diversity compared with the control unvaccinated groups (Figure 2B), and more detailed analysis revealed that this was completely driven by the unusually low alpha diversity of the water control group (Figure 2D). Compared to their respective controls, the three different vaccine doses (10^7^, 10^8^, and 10^9^ CFU/chicken) prepared in BPW and water significantly affected (*P* < 0.05) the alpha diversity of the caecal microbiota (Supplementary Figures 1A, B). However, for Marek's diluent, the vaccine doses did not significantly affect the alpha diversity (Supplementary Figure 1C). Irrespective of the three diluents, the alpha diversity was significantly higher on day 14 compared with day 7 post-vaccination (Figure 2C). The overall significant effect of vaccine dose and diluent on the alpha diversity when the data were analyzed based on vaccine diluent and dosage was mainly due to the lower diversity in the water control group (Figure 2D).

**Figure 3:**
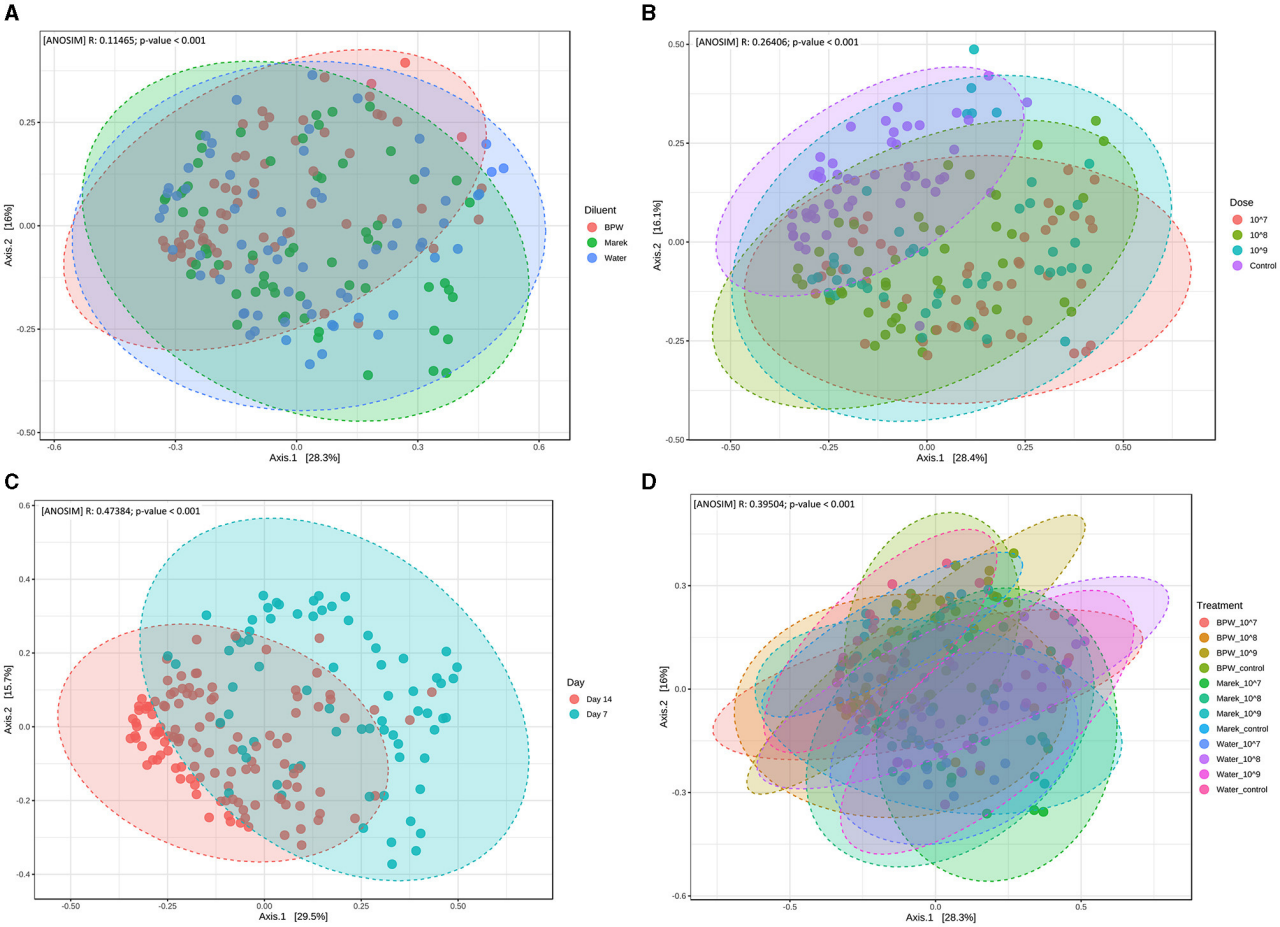
Beta diversity of the caecal microbiota of layer chickens influenced by vaccination. **(A)** Beta diversity is affected by vaccine diluent (*P* < 0.001). **(B)** Beta diversity is affected by vaccine dose (*P* < 0.001). Data are presented by pooling relevant dose treatment groups together, and the control group represents controls of BPW, Marek's, and water diluents. **(C)** Beta diversity is affected by age (*P* < 0.001). All the treatment groups were pooled together based on age. **(D)** Beta diversity is affected by vaccine dose and diluent in individual treatment groups (*P* < 0.001). Beta diversity was measured by the distance method with the Bray-Curtis index and the ANOSIM statistical method at the genus level. The abscissa (Axis 1) represents the first principal component, and the percentage represents the contribution of the first principal component to the sample difference; the ordinate (Axis 2) represents the second principal component, and the percentage represents the contribution of the second principal component to the sample difference. Each point in the Principal Coordinate Analysis (PCoA) plot represents a sample, and the distance between the points represents the dissimilarity of the caecal microbiota. Each treatment group had a minimum of six chickens at each sampling timepoint.

Beta diversity measures the distance or dissimilarity of community structure between samples. In the assessment of the effect of individual diluents and the vaccine doses, despite significant dissimilarities between the treatment groups, the beta diversity of different treatment groups overlapped with each other (Figures 3A–D). Beta diversity of the caecal microbiota was also assessed in relation to three different vaccine doses prepared in three diluents, namely, BPW, Marek's, and water. Beta diversity of the control group (unvaccinated) clustered separately (*P* < 0.001), although overlapped, compared with the groups that received 10^7^, 10^8^, and 10^9^ CFU/chick of vaccine (Figure 3B). Beta diversity was significantly affected by chicken age. Overall, beta diversity at day 7 post-vaccination was significantly different from chickens sampled at day 14 (Figure 3C). Within individual diluents (BPW, Marek's, and water), beta diversity was significantly dissimilar (*P* < 0.001), although heavily overlapped (Figure 3D). Unlike the significantly lower alpha diversity in the water control group, the beta diversity of the water control group was not significantly dissimilar from the water-vaccinated groups.

### Vaccine dose and diluent had minimal, consistent effects on the caecal microbiota

**Figure 4:**
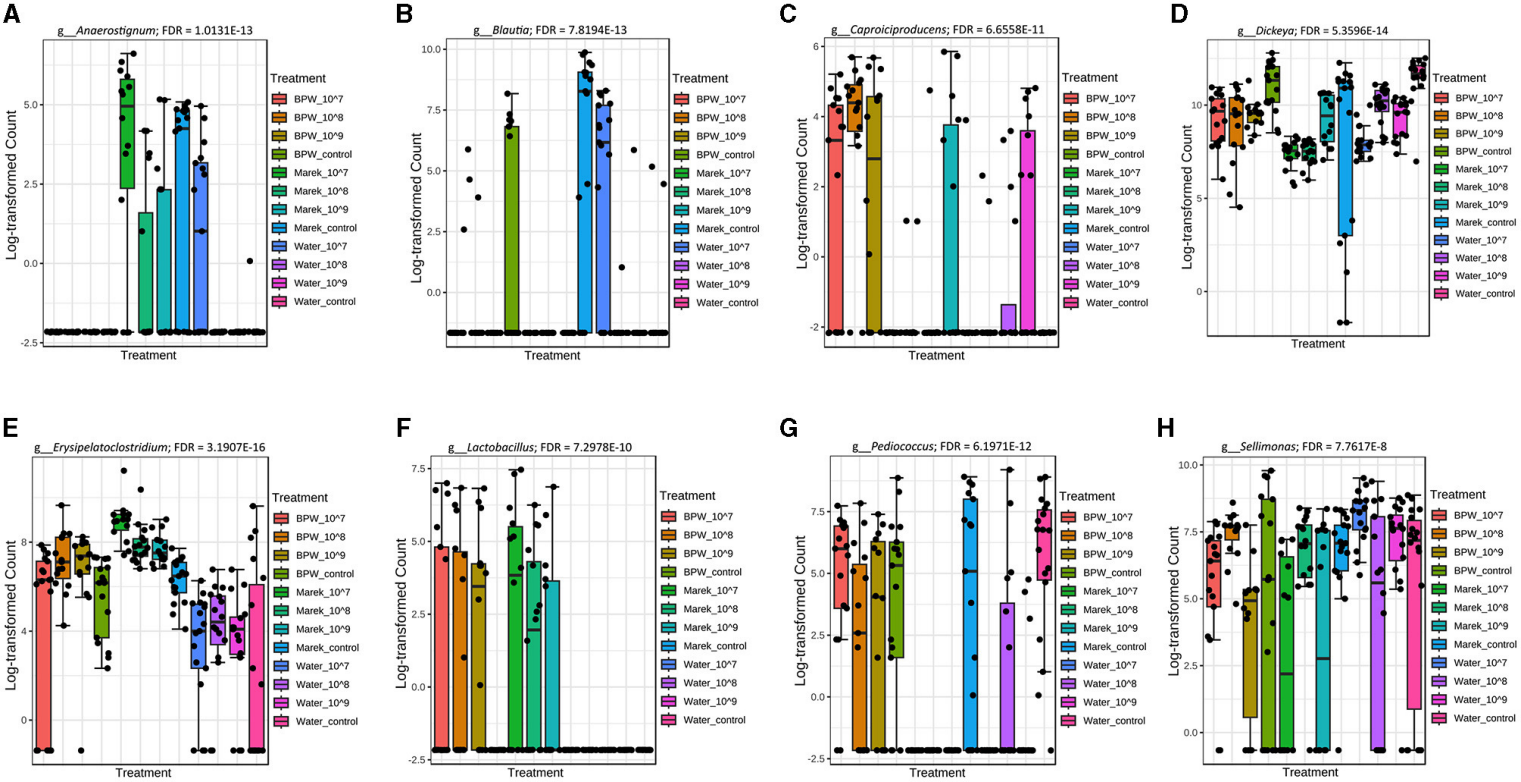
Genera that were significantly different in abundance in the various vaccine dose and diluent groups. Abundance levels of genera **(A)** *Anaerostignum* (FDR = 1.0131E-13); **(B)** *Blautia* (FDR = 7.8194E-13); **(C)** *Capriociproducens* (FDR = 6.6558E-11); **(D)** *Dickeya* (FDR = 5.3596E-14)*;* **(E)** *Erysipelatoclostridium* (FDR = 3.1907E-16); **(F)** *Lactobacillus* (FDR = 7.2978E-10); **(G)** *Pediococcus* (FDR = 6.1971E-12); and **(H)** *Sellimonas* (FDR = 7.7617E-8). Abundance levels were compared using a non-parametric test in MicrobiomeAnalyst. Each treatment group had a minimum of six chickens at each sampling timepoint.

The 16S rRNA sequencing data showed that *Anaerostignum, Blautia, Caproiciproducens, Dickeya, Erysipelatoclostridium, Lactobacillus, Pediucoccus*, and *Sellimonas* were significantly different in abundance across some vaccine doses and diluents (Figures 4A–H). The chickens inoculated with vaccine reconstituted in Marek's diluent did not display any significant alterations in the abundance of the most common genera. Only four low-abundance genera, including *Incertae*_Sedis, *Flavonifractor, Christensenellaceae*_R_7_group, and *Dickeya*, were significantly higher (FDR < 0.05) in abundance in the Marek's diluent control compared with the Marek's diluent-vaccinated groups. Interestingly, the *Blautia* abundance level was not consistent with treatments but was significantly lower in the water treatment groups (Figure 4B).

### Chicken age had more profound effects on taxa abundance in the caeca than vaccine diluent or dose

**Figure 5:**
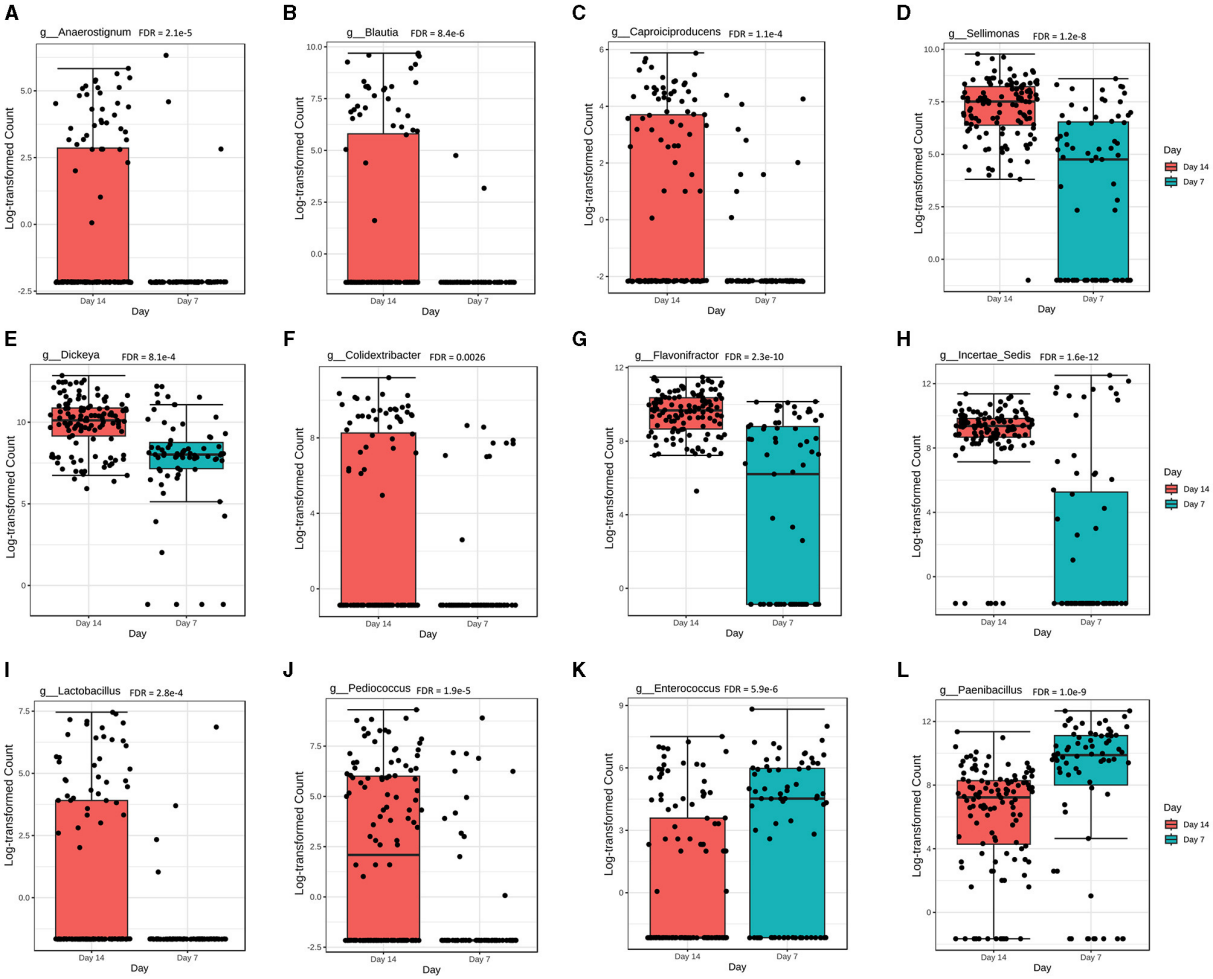
The overall abundance of caecal microbial communities is significantly affected by chicken age. Abundance levels for genera **(A)** *Anaerostignum* (FDR = 2.1e-5); **(B)** *Blautia* (FDR = 8.4e-6); **(C)** *Caproiciproducens* (FDR = 1.1e-4); **(D)** *Sellimonas* (FDR = 1.2e-8); **(E)** *Dickeya* (FDR = 8.1e-4); **(F)** *Colidextribacter* (FDR = 0.0026); **(G)** *Flavonifractor* (FDR = 2.3e-10); **(H)** *Incertae*_sedis (FDR = 1.6e-12); **(I)** *Lactobacillus* (FDR = 2.8e-4); **(J)** *Pediococcus* (FDR = 1.9e-5); **(K)** *Enterococcus* (FDR = 5.9e-6); and **(L)** *Paenibacillus* (FDR = 1.0e-9). Abundance level differences were assessed using a non-parametric test in MicrobiomeAnalyst. Each treatment group had a minimum of six chickens at each sampling timepoint.

Overall, there were 22 genera significantly different (FDR < 0.05) in abundance at day 7 compared with day 14 of chicken age. Among the 22 genera, the abundance levels of *Anaerostignum, Blautia, Caproiciproducens, Sellimonas, Dickeya, Colidextribacter, Flavonifractor, Incertae*_sedis, *Lactobacillus*, and *Pediococcus* were significantly higher at day 14 (Figures 5A–J), while *Enterococcus* and *Paenibacillus* were significantly higher at day 7 post-vaccination (Figures 5K, L).

To further understand the effects of vaccine dose and diluent, a linear discriminant analysis (LDA) effect size (LEfSe) analysis was performed on the ASV abundance data for the visualization of the top 10 most abundant genera within the caecal microbiota. LEfSe analysis was used to predict biomarkers associated with treatment groups. *Erysipelatoclostridium* was the only genera associated with Marek's diluent, while *Dickeya, Flavonifractor, Colidextribacter*, and *Clostridium*_sensu_stricto_1 were associated with BPW. *Sellimonas, Ruminococcus*_torques_group, *Propionispora*, and *Christensenellaceae*_R_7_group were associated with the water treatment group (Supplementary Figure 2A). *Flavonifractor* and UCG_005 were associated with treatment groups (all diluents combined) that received 10^9^ CFU/chick, while *Eubacterium*_hallii_group was associated with 10^8^ CFU/chick vaccine (Supplementary Figure 2B). As expected, chicken age had more profound effects, with six microbial genera associated with day 14 and four genera associated with day 7. The genera associated with day 14 samples included *Dickeya, Flavonifractor, Christensenellaceae*_R_7_group, *Incertae*_Sedis, *Sellimonas*, and *Colidextribacter* (Supplementary Figure 2C). There were not many microbial genera associated with individual vaccine doses prepared in specific diluent treatment groups (Supplementary Figure 2D).

## Discussion

In this study, the *S*. Typhimurium vaccine (Vaxsafe ST) was studied to determine if it had any effects on caecal microbiota composition. The minimum dose of Vaxsafe ST recommended by the manufacturer is 10^7^ CFU/chicken. Three different doses (10^7^, 10^8^, and 10^9^ CFUs/chicken) were included to determine whether a higher dose would affect the colonization of the vaccine in the caeca. The *S*. Typhimurium strain in Vaxsafe ST is attenuated by disruption of the *aroA* gene, whose expression in a normal *Salmonella* cell is required in the shikimate pathway for the biosynthesis of aromatic amino acids. As an auxotroph, Vaxsafe ST has reduced competitiveness compared to wild-type ST.

The average vaccine load in the caecal contents quantified through qPCR showed that the vaccine was present until day 14 post-vaccination. This shows the successful colonization ability of Vaxsafe ST for stimulation of the gut immune system in layer chickens. The qPCR data also showed that the load of vaccine significantly decreased on day 14 compared with day 7 post-vaccination. A non-significant difference in the vaccine load between the treatment groups that received the three different doses of vaccine prepared in three different diluents shows that 10^7^ CFU/chick of vaccine dose was sufficient to colonize the chicken caeca. The vaccine load data also showed that a vaccine reconstituted in water is as efficient as a vaccine reconstituted in nutritive diluents (e.g., BPW, Marek's) in gut colonization. A recent study involving AviPro *Salmonella* DUO's vaccine (a live attenuated vaccine consisting of *S*. Typhimurium and *enteritidis* strains) in commercial layer day-old chicks showed that the vaccine was not detected in cloacal swabs collected on day 2 post-vaccination (29). However, in a separate study, the vaccine strains of AviPro^®^ *Salmonella* VacE and AviPro^®^ *Salmonella* Duo were detected by culture of cloacal swabs collected 2 days post-vaccination of a day-old layer chicks (30). Vaxsafe ST could be quantified from the caeca of layer chicks at day 7 post-vaccination and could be detected in the spleen, liver, and jejunum following a culturing method (31). Perhaps differences in the detection of vaccines in various samples could be due to multiple factors, including the sensitivity of the tests applied. Overall, the qPCR data of Vaxsafe ST quantification from caecal contents show that the vaccine colonized the gut effectively and was quantifiable for at least 14 days post-vaccination, which was the experimental period of the current study.

The objective of reconstituting Vaxsafe ST in nutritive diluents (BPW and Marek's) was to determine whether these diluents could improve vaccine colonization in the chicken caeca. Water was used as a control, as it is the diluent used for administering Vaxsafe ST to chicks using a coarse spray. Additionally, it was aimed at establishing whether the dose and diluent could cause a significant shift in the structure of the caecal microbiota of layer chicks. The dosage of the vaccine (10^7^, 10^8^, and 10^9^ CFU/chick) did not significantly change the composition of the gut microbiota. Statistically significant differences in caecal microbiota beta diversity were found between vaccinated and unvaccinated chickens, but the changes driving that difference were mainly among low-abundance taxa and were not shared across different diluent groups. The dose and diluent did not significantly change the colonization of the vaccine in the gut, suggesting that the recommended 10^7^ CFUs/chick dose during vaccination is appropriate. However, in contrast, a recent study using a dose of 10^9^ CFU/chicken of a different recombinant attenuated *Salmonella* vaccine (*S*. Typhimurium strain UK-1) has shown a major shift in the diversity of the caecal microbiota of the vaccinated chickens (19). A previously published study has shown that the AviPro *Salmonella* Duo (which contains *S*. *enteritidis* and *S*. Typhimurium serovars) vaccine in specific pathogen-free layer chickens (< 16 days old) did not significantly affect the alpha diversity of the caecal microbiota (32).

The analysis showed that overall the vaccine reconstituted in three different diluents did not change the alpha diversity of the caecal microbiota. Marek's vaccine diluent contains peptone and sucrose as the main ingredients, in addition to salts. Overall, a higher alpha diversity at day 14 compared with day 7 of the chicken's age showed that the population of caecal microbial communities in individual chickens became more diverse as the chickens aged. A higher alpha diversity of caecal microbiota at day 12 compared with days 5, 3, and 0 of layer chicken age has been previously reported (9, 33). Overall, the data showed that Vaxsafe ST administration minimally affected the alpha diversity of the caecal microbiota of layer chicks.

In the current study, a significantly dissimilar beta diversity at day 14 compared with day 7 post-vaccination showed that the caecal microbiota changed with chicken age. A shift in the beta diversity of the gut microbiota with chicken age has been previously reported (7). In the current study, an overlap in the beta diversity of different treatment groups showed that the *Salmonella* vaccination only minimally changed the community structure of the caecal microbiota. A previous study in 5-week-old layer chickens showed that a different *Salmonella* vaccine did change the composition of the gut microbiota in terms of beta diversity (19).

It is important to evaluate the impact of Vaxsafe ST on gut microbiota composition and understand that it is quite different from the effect of infection with wild-type *Salmonella* on chicken gut microbiota. Studies have shown that wild-type *Salmonella* infections negatively impact alpha and beta diversities of the gut microbiota, leading to a population surge of *Enterobacteriaceae*. For example, *S*. Typhimurium infection in 1-week-old layer chickens significantly changed the alpha and beta diversities of the caecal microbiota; decreased the abundance of *Coprococcus, Ruminococcus, Lactococcus*, and *Lactobacillus*; and increased unclassified *Enterobacteriaceae* (34). *Salmonella enteritidis* infection in a week-old layer of chickens significantly reduced overall diversity by increasing the abundance level of *Enterobacteriaceae* (35). A higher abundance of *Lactobacillus* in the BPW and Marek's diluents administered to vaccine groups shows the usefulness of nutrient-rich diluents used for vaccine reconstitution. The chicken age effect was obvious in the abundance of various microbial communities. Overall, *Anaerostignum, Blautia, Caproiciproducens, Dickeya, Lactobacillus, Pediococcus, Sellimonas*, and *Colidextribacter* significantly increased in abundance on day 14 compared with day 7 of chicken age. Among these communities, *Lactobacillus* has been shown, in a previous study, to be low on day 1 compared with days 7, 21, and 35 of broiler age (36). *Blautia* is involved in the production of short-chain fatty acids, primarily acetate (37), and plays a positive role in the modulation of gut functions (38). *Lactobacillus* has been widely implicated in the gut health of chickens (39, 40). In the future, vaccine manufacturers might use probiotics during vaccination to further improve the gut microbial environment as part of their gut health strategy. Studies in layer chickens have shown that both the *Salmonella* vaccine and probiotics are useful in improving gut health (19, 41).

Data obtained in this study demonstrate that Vaxsafe ST shows good levels of caecal colonization with a dose of 10^7^ CFU/chick without disruption of the caecal microbiota. The vaccine can be used with confidence, knowing that there is less risk that it can cause dysbiosis.

## Data availability statement

The original contributions presented in the study are publicly available. This data can be found here: https://www.ncbi.nlm.nih.gov/bioproject/; PRJNA1003953.

## Ethics statement

The animal study was approved by the University of Adelaide Animal Ethics Committee. The study was conducted in accordance with the local legislation and institutional requirements.

## Author contributions

SK: Data curation, Formal analysis, Investigation, Methodology, Validation, Visualization, Writing – original draft. AM: Conceptualization, Investigation, Methodology, Project administration, Resources, Writing – review & editing. DA: Conceptualization, Funding acquisition, Project administration, Writing – review & editing. GU: Conceptualization, Funding acquisition, Project administration, Supervision, Writing – review & editing. RM: Investigation, Project administration, Resources, Supervision, Writing – review & editing. TV: Investigation, Methodology, Resources, Writing – review & editing. RG: Investigation, Project administration, Supervision, Visualization, Writing – review & editing. KC: Conceptualization, Funding acquisition, Investigation, Methodology, Project administration, Resources, Supervision, Writing – review & editing.
