# Supplementary material for: A live attenuated Salmonella Typhimurium vaccine dose and diluent have minimal effects on the caecal microbiota of layer chickens
Source: Front Vet Sci. 2024 Apr 15;11:1364731. doi: 10.3389/fvets.2024.1364731 (PMC11057240; doi:10.3389/fvets.2024.1364731)
Supplement: Supplementary file 1 [file Data_Sheet_1.docx]

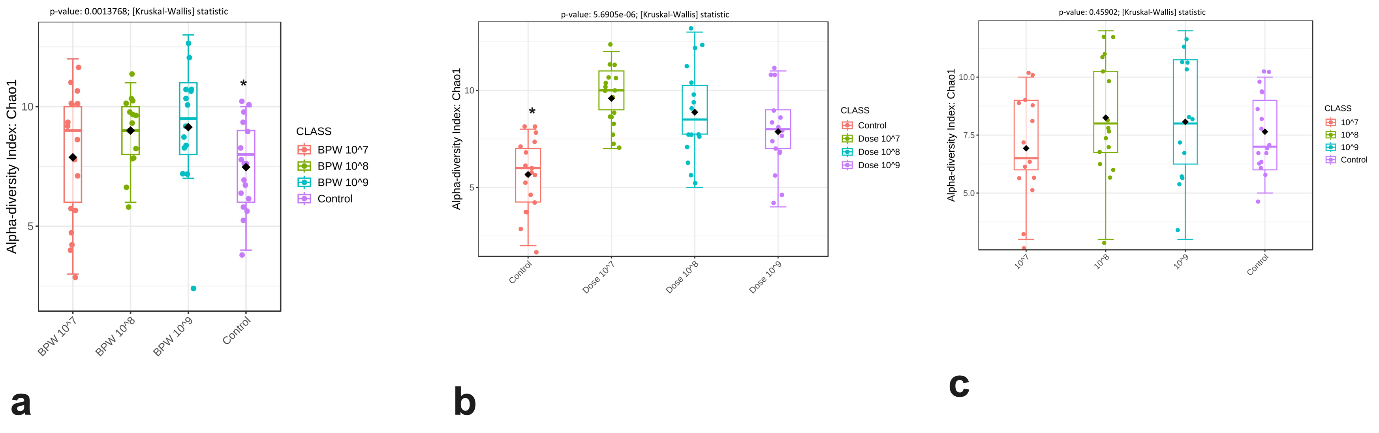


Supplementary Figure 1: Alpha diversity of chicks affected by vaccine dose within individual diluent treatment groups. **a)** Vaccine dose effects in BPW diluent treatment groups. **b)** Vaccine dose effects in water diluent treatment groups. **c)** Vaccine dose effects in Marek diluent treatment groups. Asterisk (*) within each graph shows significant difference from the other treatment groups.


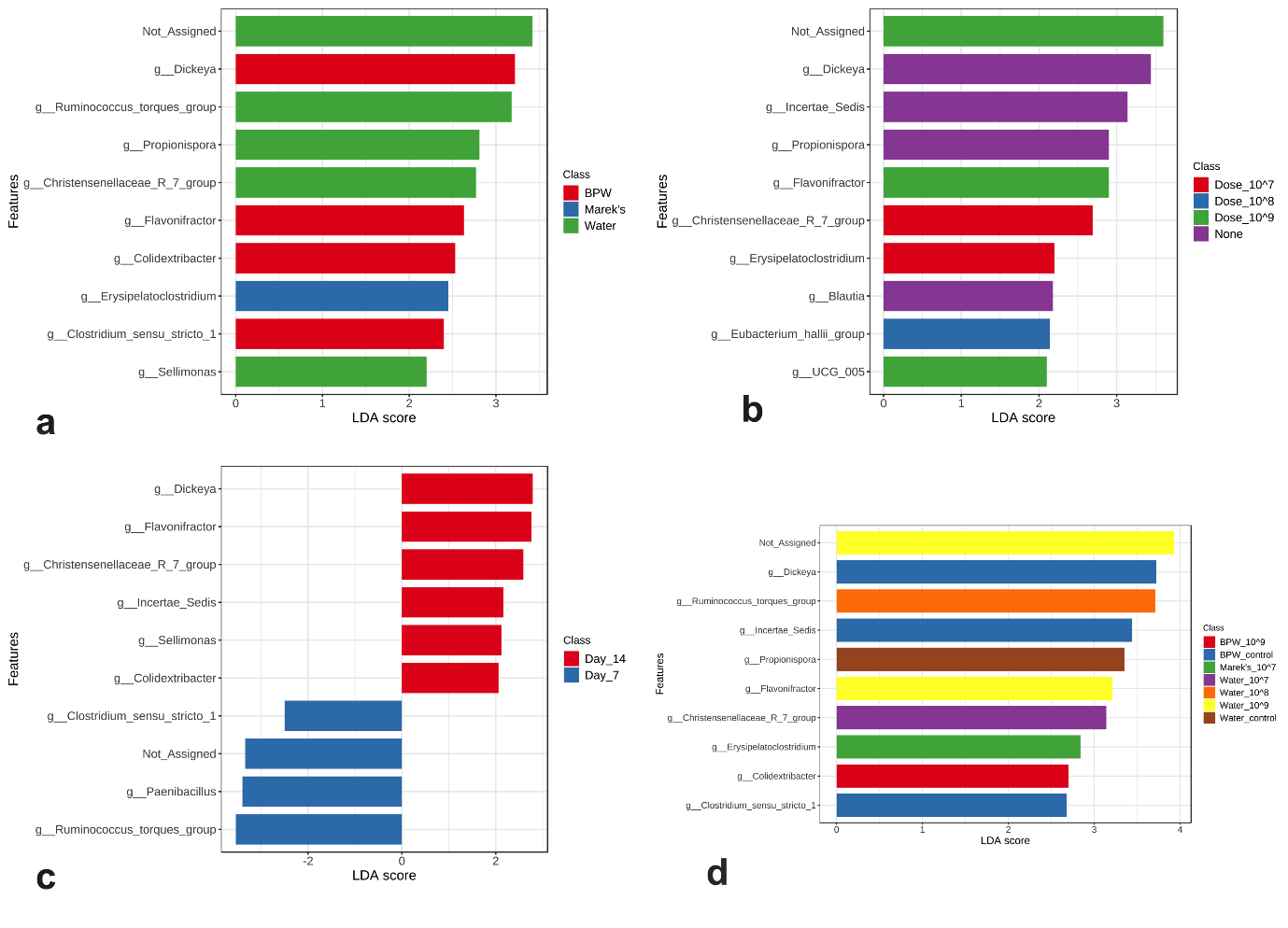


Supplementary Figure 2: Linear discriminant analysis (LDA) effect size (LEfSe) analysis showing bacterial genera affected by vaccine diluent, dose and chicken age. a) Bacterial genera specific to vaccine diluent. b) Bacterial genera affected by vaccine dose. C) Bacterial genera affected by chicken age. d) Microbial genera affected by vaccine dose in BPW and water treatment groups.
