# Supplementary material for: A live attenuated Salmonella Typhimurium vaccine dose and diluent have minimal effects on the caecal microbiota of layer chickens
Source: Front Vet Sci. 2024 Apr 15;11:1364731. doi: 10.3389/fvets.2024.1364731 (PMC11057240; doi:10.3389/fvets.2024.1364731)
Supplement: Supplementary file 2 [file Table_1.DOCX]

**Table 1.** Treatment group details of Isa-Brown layer chicks for Vaxsafe ST vaccination and characterisation of caecal microbiota

| Treatment | Vaccine reconstitution medium | Vaccination dose (CFU/chick) |
| --- | --- | --- |
| NC Marek’s | Marek’s diluent | Nil |
| Marek’s 10^7^ | Marek’s diluent | 10^7^ |
| Marek’s 10^8^ | Marek’s diluent | 10^8^ |
| Marek’s 10^9^ | Marek’s diluent | 10^9^ |
| NC BPW | Buffered peptone water control | Nil |
| BPW 10^7^ | Buffered peptone water | 10^7^ |
| BPW 10^8^ | Buffered peptone water | 10^8^ |
| BPW 10^9^ | Buffered peptone water | 10^9^ |
| NC water | Water control | Nil |
| Water 10^7^ | Water | 10^7^ |
| Water 10^8^ | Water | 10^8^ |
| Water 10^9^ | Water | 10^9^ |

From each treatment group, 6 chickens were culled on day 7 and up to 11 chicks on day 14 post-vaccination for 16S rRNA gene amplicon characterisation of caecal microbiota and vaccine load determination by qPCR. NC = negative control.
